# Supplementary material for: Association of Public Sports Space Perception with Health-Related Quality of Life in Middle-Aged and Older Adults—Evidence from a Survey in Shandong, China
Source: Behav Sci (Basel). 2023 Sep 4;13(9):736. doi: 10.3390/bs13090736 (PMC10525232; doi:10.3390/bs13090736)
Supplement: Supplementary file 1 [file behavsci-13-00736-s001.zip › behavsci-2554561-supplementary.pdf]

Supplementary Material

Supplement 1:

Table S1: The characteristic of the study population (N = 869).

Variables		Number	Percent(%)
Sex	Male	401	46.19
	Female	468	53.81
Living situation	Live by yourself	244	28.06
	Only live with children	507	58.38
	Only live with their parents	107	12.29
	While living with their children and their parents	11	1.27
living environment	rural area	350	40.30
	city	387	44.57
	town center	41	4.73
	Urban and rural areas or township combined areas	87	10.05
	special area	3	0.35
The type of community	Native community	267	30.71
	Restructuring community	50	5.73
	New community	201	23.18
	Village and town self-built houses	317	36.53
	Other types	33	3.85
Smoking	Quit smoking	97	11.20
	Always smoking	143	16.51
	Never smoked	628	72.29
Drinking	more than once a month	265	30.48
	Less than once a month	135	15.59
	Don't drink anything	469	53.93
Disease	0	409	47.11
	1	179	20.55
	2	106	12.24
	3	72	8.31
	≥4	40	4.62

Supplement 2:

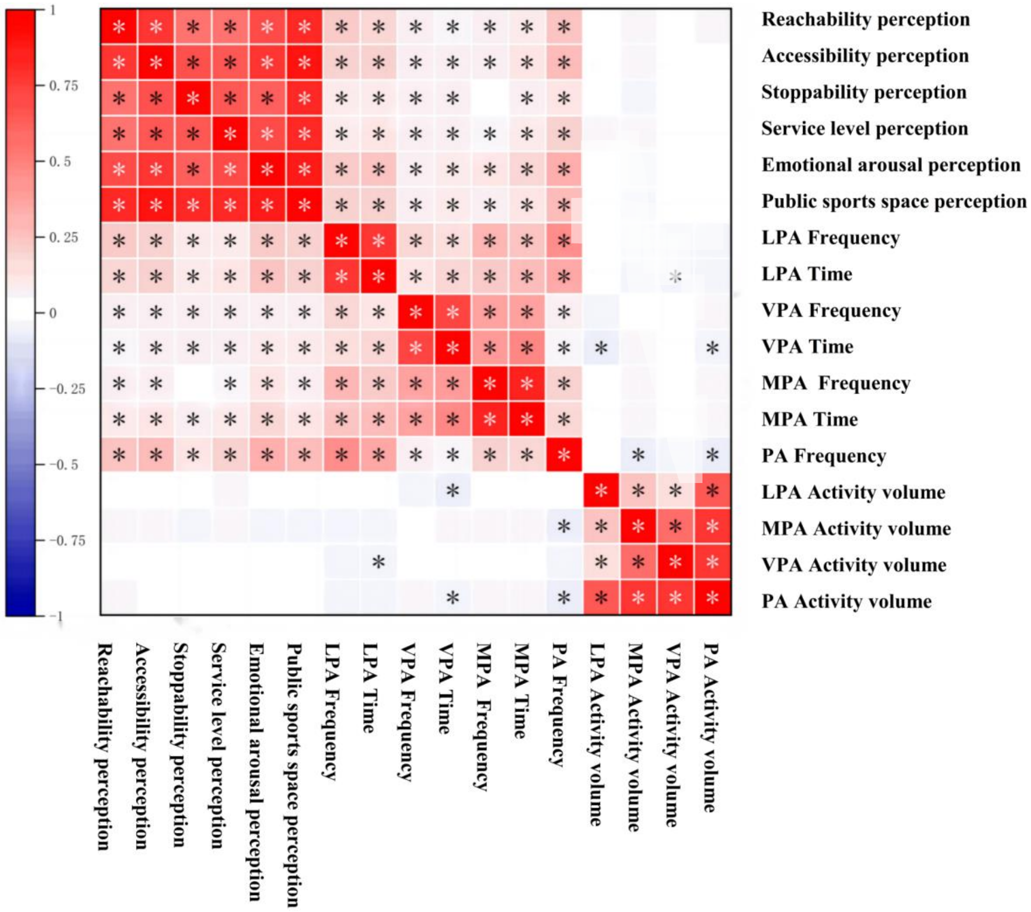

Figure S1: Correlation analysis of public sports space perception and physical activity-related variables.*p<0.001. Light-intensity PA (LPA), moderate-intensity PA (MPA), vigorous-intensity PA (VPA).

Supplement 3:

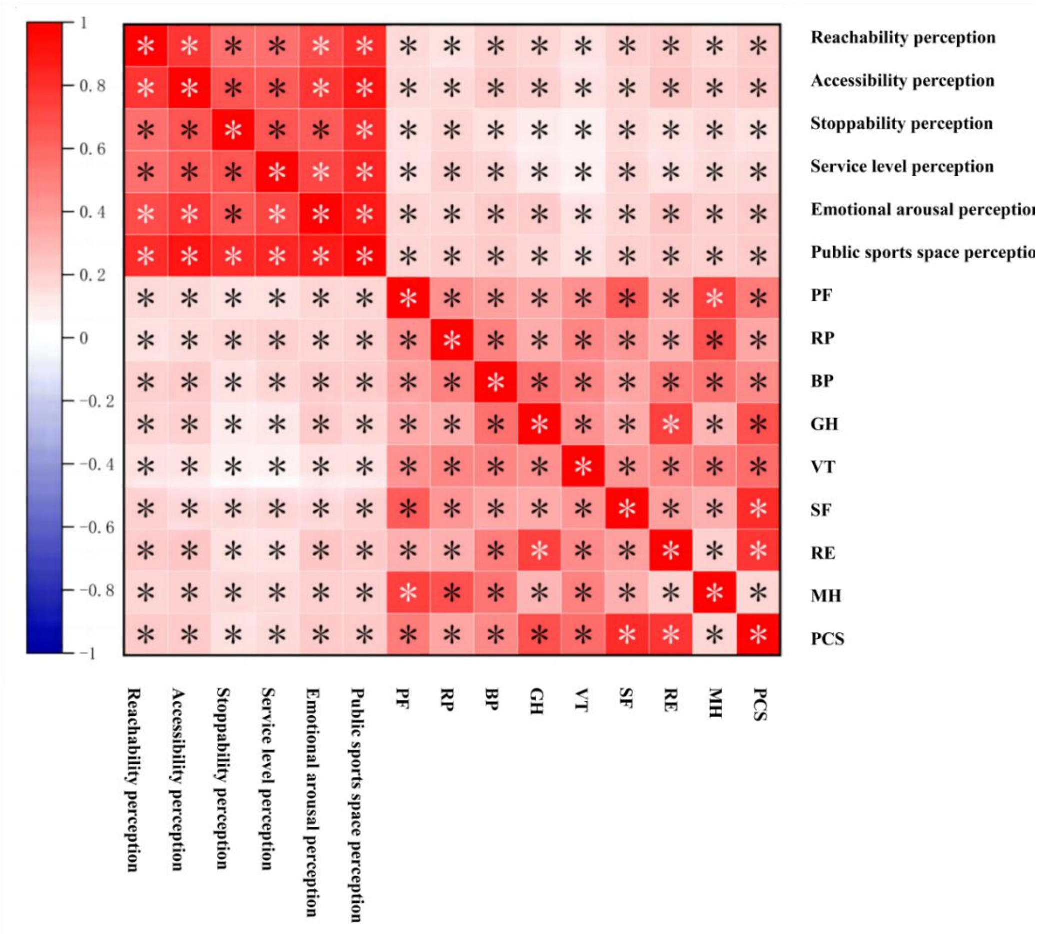

Figure S2: Correlation analysis of public sports space perception and variables related to health-related quality of life.*p<0.001.physical function (PF), role–body (RP), body pain (BP), general health (GH), vitality (VT), social functioning (SF), role–emotion (RE), and mental health (MH).

Supplement 4:

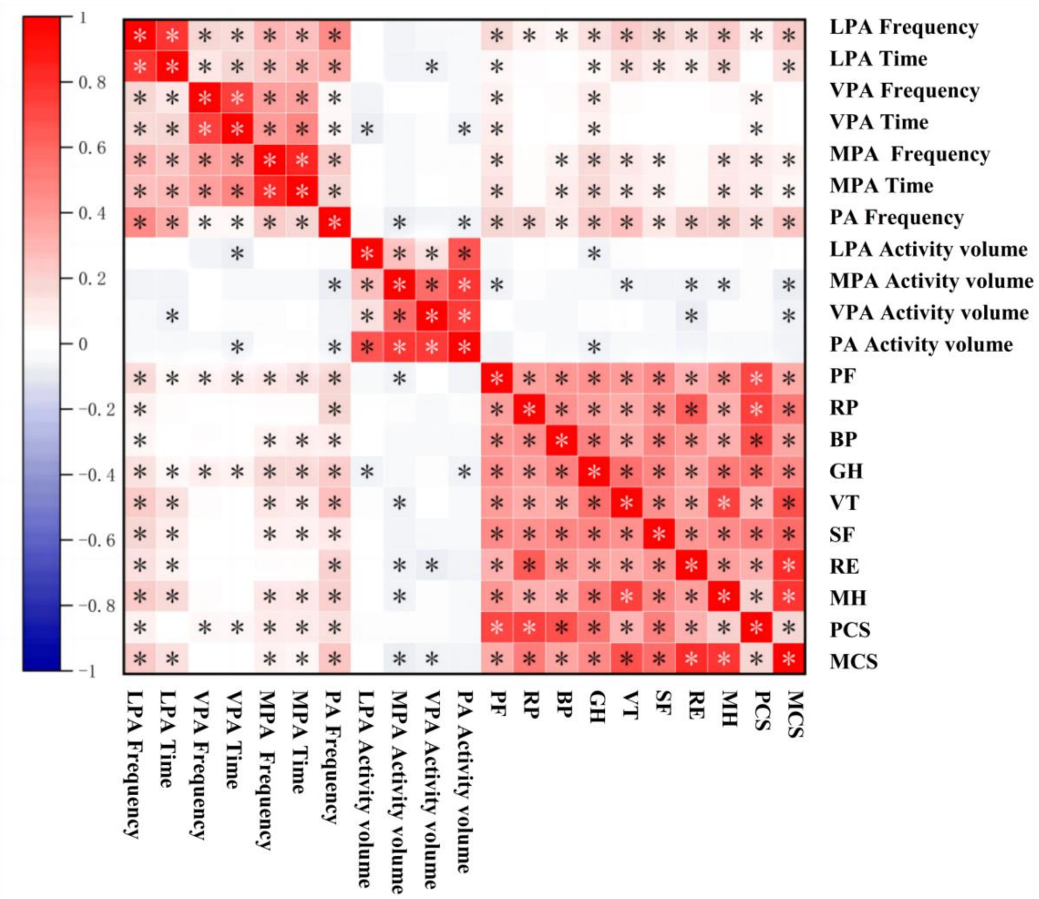

Figure S3: Correlation analysis of physical exercise activity and health-related QoL-related variables.*p<0.001. physical function (PF), role–body (RP), body pain (BP), general health (GH), vitality (VT), social functioning (SF), role–emotion (RE), and mental health (MH). Light-intensity PA (LPA), moderate-intensity PA (MPA), vigorous-intensity PA (VPA).

Supplement 5:

Table S2: Regression analysis of public sports space perception and analysis of various dimensions on physical activity.

Variables		Reachability perception	Accessibility perception	Stoppability perception	Service level perception	Emotional arousal perception	Public sports space perception
Time	LPA	-0.003	0.009	-0.012	-0.035**	0.054**	0.013**
	VPA	-0.01	0.001	-0.004	-0.005	0.02	0.003*
	MPA	-0.006	-0.008	-0.019	-0.001	0.038**	0.005**
Frequency	LPA	0.059	-0.007	-0.043	-0.117**	0.138**	0.030**
	VPA	0.001	-0.005	-0.009	-0.001	0.019	0.005*
	MPA	0.009	-0.034	-0.03	-0.009	0.070**	0.006*
Activity volume	LPA	0.004	-0.002	0.015	-0.017	0.001	0.001
	VPA	-0.026	0.029	0.001	0.006	-0.01	0.003
	MPA	-0.001	0.014	-0.005	0.008	-0.019	-0.003
	All	0.006	0.011	-0.043**	-0.032*	0.078**	0.019**

* p<0.05, ** p<0.01.

Supplement 6:

Table S3: Regression analysis of public sports space perception and each dimension of health-related quality of life and its various dimensions.

Variables	Reachability perception	Accessibility perception	Stoppability perception	Service level perception	Emotional arousal perception	Public sports space perception
PF	0.437	-0.164	-0.213	-0.826**	0.979**	0.213**
RP	0.333	-0.565	-0.331	0.007	0.814	0.259**
BP	-0.235	-0.366	0.044	0.297	0.392	0.132**
GH	0.125	-0.066	-0.665**	0.110	0.662*	0.166**
VT	-0.096	0.301	-0.558**	-0.280	0.768**	0.136**
SF	0.092	0.162	-0.342	-0.436	0.621	0.097**
RE	0.838	-0.495	0.102	0.261	-0.387	0.319**
MH	0.109	0.273	-0.350	-0.466*	0.599*	0.166**
PCS	0.014	-0.166	-0.103	-0.064	0.377**	0.059**
MCS	0.211	0.092	-0.154	-0.150	0.132	0.131**

* $p<0.05$, ** $p<0.01$.physical function (PF), role–body (RP), body pain (BP), general health (GH), vitality (VT), social functioning (SF), role–emotion (RE), and mental health (MH).

Supplement 7:

Table S4: Regression analysis of physical exercise activities on health-related quality of life and its various dimensions.

Variables	Time			Frequency			all
	LPA	VPA	MPA	LPA	VPA	MPA	
PF	-1.254	2.477	1.489	1.618**	-0.266	-1.314	2.724**
RP	1.004	1.148	-2.689	0.132	-0.774	0.548	4.783**
BP	0.191	2.985*	-2.968	0.357	-0.48	0.534	1.362*
GH	-0.694	1.767	-0.506	0.669	0.646	0.061	3.132**
VT	-0.112	0.861	-1.751	0.932**	0.227	0.085	2.760**
SF	-0.169	1.393	-2.189*	1.441**	0.071	-0.179	1.044
RE	-0.639	0.39	-0.605	1.269	-0.533	-0.171	4.195**
MH	0.561	0.844	-3.341*	0.780*	0.565	0.608	1.562**
PCS	-0.151	1.119*	-0.11	0.232*	-0.254	-0.14	1.109**
MCS	0.083	0.219	-1.709	0.715**	0.133	0.231	1.526**

* $p<0.05$, ** $p<0.01$.physical function (PF), role–body (RP), body pain (BP), general health (GH), vitality (VT), social functioning (SF), role–emotion (RE), and mental health (MH).

Supplement 8:

1.Variance Analysis

1.1 Analysis of perceived age differences in public sports space among community elderly people

The results of variance analysis showed that there were significant differences in accessibility perception, suitability perception and sports space perception among different age groups, with p-values of 0.005, 0.000 and 0.035, respectively. This means that there are obvious differences in the perception of accessibility, stoppability and overall perception of public sports space among respondents in different age groups. The p-values of reachability, emotional arousal perception and service level perception were all greater than 0.05, indicating that there was no significant difference in the perception scores of service level perception among different age groups.

Table S5: The current situation of public sports space perception for the middle-aged and older adults

Variables	Age（Mean±SD）				F	p
	All	40-59	60-69	70-80		
Reachability perception	3.679±0.975	3.641±0.983	3.683±0.968	3.585±1.113	1.921	0.125
Accessibility perception	3.689±0.952	3.647±0.947	3.688±0.963	3.508±1.040	4.336	0.005**
Stoppability perception	3.405±1.052	3.398±1.107	3.288±1.033	3.055±0.905	7.727	0.000**
Service level perception	3.280±1.079	3.213±1.105	3.355±1.050	3.090±1.045	2.368	0.069
Emotional arousal perception	3.656±0.954	3.618±0.951	3.688±0.970	3.700±1.005	0.261	0.853
Public sports space perception	3.542±0.864	3.503±0.872	3.541±0.878	3.388±0.835	2.883	0.035*

* p<0.05, ** p<0.01.

1.2 The status and age difference of the elderly in the community

The results of variance analysis showed that there were significant differences in the participation time of LPA (p<0.01) and VPA (p<0.01) among middle-aged and older adults people in different age groups. The mean values of participation time of LPA for the early old age group aged 40-59, the middle-aged and older adults group aged 60-69, and the middle-aged and older adults group aged 70-80 were 1.820, 2.361, and 2.283, respectively. The mean values of participation time of VPA for the three groups were 1.211, 1.095, and 1.019, respectively. However, there was no significant difference in the participation time of MPA among middle-aged and older adults people in different age groups (p>0.05). In terms of participation frequency, there were significant differences in the participation frequency of LPA (p<0.01) and VPA (p<0.01) among middle-aged and older adults in different age groups. The mean values of participation frequency of LPA for the three groups were 1.960, 3.120, and 3.179, respectively. The mean values of participation frequency of VPA for the three groups were 0.411, 0.177, and 0.066, respectively. However, there was no significant difference in the participation frequency of MPA among middle-aged and older adults in different age groups (p>0.05). In terms of physical exercise activity volume, there were significant differences in the activity volume of LPA (p<0.01) among middle-aged and older adults in different age groups. The mean values of activity volume of LPA for the three groups were -0.121, 0.431, and 0.486, respectively. The mean values of activity volume of VPA for the three groups were 0.054, -0.099, and -0.170, respectively. However, there was no significant difference in the

activity volume of MPA among middle-aged and older adults in different age groups (p>0.01). Generally speaking, there were significant differences in the total physical exercise activity volume among middle-aged and older adults in different age groups (p<0.05).

Table S6: Current situation and age difference of physical activities

Variables		Age（Mean±SD）				F	p
		All	40-59	60-69	70-80		
Time	LPA	1.976±1.113	1.82±1.028	2.361±1.217	2.283±1.217	20.212	0.000**
	VPA	1.166±0.593	1.211±0.661	1.095±0.462	1.019±0.194	6.222	0.002**
	MPA	1.266±0.751	1.268±0.744	1.323±0.824	1.170±0.669	1.325	0.266
frequency	LPA	2.321±2.776	1.960±2.577	3.120±2.959	3.179±3.153	17.324	0.000**
	VPA	0.326±1.187	0.411±1.329	0.177±0.778	0.066±0.680	5.374	0.005**
	MPA	0.517±1.505	0.502±1.453	0.677±1.716	0.358±1.449	1.514	0.221
activity volume	LPA	0.054±1.028	-0.121±0.819	0.431±1.240	0.486±1.410	30.660	0.000**
	VPA	-0.002±0.995	0.054±1.133	-0.099±0.629	-0.170±0.395	3.238	0.040*
	MPA	0.025±1.053	0.017±1.061	0.093±1.086	-0.031±0.964	0.498	0.608
	All	0.035±1.024	-0.026±1.069	0.196±0.966	0.142±0.802	3.616	0.027*

* p<0.05, ** p<0.01.

1.3 Status and age differences of health-related quality of life among the elderly in the community

The results of variance analysis showed that there were significant differences in physical function, bodily pain, general health, social function and body composition score among middle-aged and older adults in different age groups (p<0.01). The mean values of physical function for the early old age group aged 40-59, the middle-aged and older adults group aged 60-69, and the middle-aged and older adults group aged 70-80 were 83.552, 69.185, and 63.548, respectively. The mean values of bodily pain for the three groups were 84.268, 78.232, and 70.710, respectively. The mean values of general health for the three groups were 71.539, 63.446, and 61.032, respectively. The mean values of social function for the three groups were 83.346, 79.403, and 70.645, respectively. The mean values of body composition score for the three groups were 53.230, 48.378, and 46.032, respectively. However, there was no significant difference in role physical, vitality, role emotional, mental health and psychological component score among middle-aged and older adults people in different age groups (p>0.05). Generally speaking, the age difference was more reflected in the physical performance of health-related quality of life, and the impact on psychology was not significant.

Table S7: Status and age differences of health-related quality of life

Variables	Age（Mean±SD）				F	p
	All	40-59	60-69	70-80		
PF	78.990±21.309	83.552±18.801	69.185±23.002	63.548±22.516	52.087	0.000**
RP	81.611±34.721	83.153±33.759	78.326±36.142	75.806±41.071	2.072	0.127
BP	82.177±18.883	84.268±17.819	78.232±19.658	70.710±24.243	14.951	0.000**
GH	68.986±19.975	71.539±19.912	63.446±19.211	61.032±16.903	16.909	0.000**
VT	69.417±17.071	69.925±17.563	68.305±15.86	67.258±15.960	1.008	0.365
SF	81.742±19.879	83.346±19.132	79.403±20.245	70.645±26.005	8.532	0.000**
RE	81.255±35.064	0.054±1.028	-0.121±0.819	0.431±1.240	0.095	0.910
MH	70.314±16.132	70.589±16.675	69.906±14.683	68.516±16.19	0.353	0.702
PCS	51.691±8.308	53.230±7.465	48.378±8.888	46.032±9.607	39.301	0.000**
MCS	43.01±12.994	42.709±13.359	43.867±12.156	42.581±11.837	0.685	0.504

* p<0.05, ** p<0.01.physical function (PF), role–body (RP), body pain (BP), general health (GH), vitality (VT), social functioning (SF), role–emotion (RE), and mental health (MH).
